# Laboratory three-dimensional X-ray micro-beam Laue diffraction

**DOI:** 10.1107/S1600576725007587

**Published:** 2025-09-24

**Authors:** Yubin Zhang, Anthony Seret, Jette Oddershede, Azat Slyamov, Jan Kehres, Florian Bachmann, Carsten Gundlach, Ulrik Lund Olsen, Jacob Bowen, Henning Friis Poulsen, Erik Lauridsen, Dorte Juul Jensen

**Affiliations:** a Department of Civil and Mechanical Engineering, Technical University of Denmark, 2800Kongens Lyngby, Denmark; b Observatoire de la Côte d’Azur, CNRS, Laboratoire Lagrange, Université Côte d’Azur, CS 34229 – F06304 Nice Cedex 4, France; c Xnovo Technology ApS, 4600Køge, Denmark; d Department of Physics, Technical University of Denmark, 2800Kongens Lyngby, Denmark; Montanuniversität Leoben, Austria

**Keywords:** three dimensional X-ray diffraction, 3DXRD, laboratory diffraction contrast tomography, Laue micro-beam diffraction, scanning 3DXRD, intragranular orientation

## Abstract

The development of 3D non-destructive X-ray characterization techniques in home laboratories is essential for enabling many more researchers to perform 3D characterization daily, overcoming the limitations imposed by competitive and scarce access to synchrotron facilities. Recent efforts have focused on techniques such as laboratory diffraction contrast tomography (LabDCT). LabDCT allows 3D characterization of recrystallized grains with sizes larger than 15–20 µm, offering a boundary resolution of approximately 5 µm using commercial X-ray computed tomography (CT) systems. To enhance the capa­bil­ities of laboratory instruments, we have developed a new laboratory-based 3D X-ray micro-beam diffraction (Lab-3DµXRD) technique. Lab-3DµXRD combines the use of a focused polychromatic beam with a scanning-tomographic data acquisition routine to enable depth-resolved crystallographic orientation characterization. This work presents the first realization of Lab-3DµXRD, including hardware development through the integration of a newly developed Pt-coated twin paraboloidal capillary X-ray focusing optics into a conventional X-ray micro-computed tomography (µCT) system, as well as the development of data acquisition and processing software. The results are validated through comparisons with LabDCT and synchrotron phase contrast tomography. The findings clearly demonstrate the feasibility of Lab-3DµXRD, particularly in detecting smaller grains and providing intragranular information. Finally, we discuss future directions for developing Lab-3DµXRD into a versatile tool for studying materials with smaller grain sizes and high defect densities, including the potential of combining it with LabDCT and µCT for multiscale and multimodal microstructural characterization.

## Abstract 2

A novel laboratory-based 3D X-ray micro-beam diffraction technique has been developed and successfully validated. The setup enables the detection of grains as small as 10 µm, with an intragranular orientation uncertainty of 0.01°.

## 1. Introduction

Advances in the characterization of crystalline materials are essential for unraveling their processing–structure–property relationships, guiding the development of next-generation materials with tailored properties. Among the many techniques available, 3D non-destructive grain mapping using X-rays has emerged as a cornerstone for gaining detailed insights into the internal microstructure, local crystallographic orientations and defect distributions of materials (Juul Jensen & Poulsen, 2000 ▸; Offerman *et al.*, 2002 ▸; King *et al.*, 2008 ▸; Barabash *et al.*, 2009 ▸; Ice *et al.*, 2011 ▸; Juul Jensen & Poulsen, 2012 ▸; Pokharel *et al.*, 2014 ▸; Juul Jensen & Zhang, 2020 ▸; Shahani *et al.*, 2020 ▸). Broadly, these techniques can be categorized into two families: those using monochromatic beams, such as 3D X-ray diffraction (3DXRD) (Poulsen, 2004 ▸; Johnson *et al.*, 2008 ▸; Li *et al.*, 2012 ▸); and those using polychromatic beams, such as Laue 3D micro-beam X-ray diffraction (3DµXRD) (Larson *et al.*, 2002 ▸).

3DXRD typically employs a monochromatic beam and a tomographic data acquisition to map grain orientations and strain fields in three dimensions. With a line or a box beam, a large portion of or even an entire grain is illuminated by the beam, limiting the capability of the method to resolve intragranular information (Li *et al.*, 2012 ▸; Pokharel *et al.*, 2015 ▸; Viganò *et al.*, 2016 ▸; Winther *et al.*, 2017 ▸). To improve spatial resolution, focused-beam configurations and scanning methods have been introduced, leading to the development of scanning 3DXRD (S3DXRD) (Hayashi *et al.*, 2019 ▸; Wright *et al.*, 2020 ▸; Li *et al.*, 2023 ▸; Henningsson *et al.*, 2024 ▸). 3DµXRD uses a focused polychromatic beam combined with differential aper­ture scanning to achieve depth-resolved orientation indexing, avoiding the need for sample rotation (Larson & Levine, 2013 ▸). After 20 years of development, these techniques have become indispensable tools in materials science, offering superior resolutions: spatial (50–100 nm at best), angular (0.005–0.01°) and strain sensitivity (0.5–1 × 10^−4^). Nonetheless, synchrotron-based methods are inherently limited by the accessibility of large-scale facilities – extended measurement times (>1 week) are not realistic, and the turn­around time (from idea to result) is very long, typically a year or more.

To address these limitations, significant efforts have been directed toward adapting advanced X-ray characterization methods in home laboratories. One such method, laboratory X-ray diffraction contrast tomography (LabDCT), has become an established 3D characterization tool (King *et al.*, 2013 ▸; Feser *et al.*, 2015 ▸; McDonald *et al.*, 2015 ▸; Holzner *et al.*, 2016 ▸; Oddershede *et al.*, 2022 ▸). LabDCT utilizes a conical polychromatic X-ray beam generated from laboratory X-ray tubes in conventional X-ray computed tomography (CT)systems, combined with diffraction principles based on the Laue focusing effect (Kvardakov *et al.*, 1997 ▸; Guinier & Tennevin, 1949 ▸). It enables the 3D mapping of grain orientations and morphologies without requiring access to synchrotron facilities. However, even when using all the flux from the entire polychromatic X-ray spectrum from laboratory X-ray tubes, it remains challenging to use this technique to map grains smaller than 20 µm (Fang *et al.*, 2021 ▸; Fang *et al.*, 2023 ▸). Like its synchrotron predecessor, diffraction contrast tomography – a variation of 3DXRD – the use of a large beam limits its application for studying deformed materials with intragranular orientation or strain variations. Additionally, the polychromatic beam makes the study of deformed samples even more challenging.

At the same time, laboratory micro-beam diffraction (Lab-µXRD) setups in transmission geometry have been developed, enabling the characterization of intragranular information in thin samples (Lynch *et al.*, 2007 ▸; Lynch *et al.*, 2019 ▸; Zhang *et al.*, 2024 ▸). However, so far, this technique has only been used for 2D mapping. This is probably because of the weak beam intensity in laboratory sources, which makes techniques like the differential aperture method (Larson *et al.*, 2002 ▸) – commonly used at synchrotrons for depth-resolved (the third dimension) orientation indexing and strain fitting – impractically long for 3D mapping in laboratory settings.

To enable high-resolution 3D mapping in the laboratory, we introduce a new method that combines the principles of S3DXRD and Lab-µXRD: laboratory 3D micro-beam X-ray diffraction (Lab-3DµXRD). Specifically, we propose to combine the use of a focused polychromatic beam (inspired by µXRD) and the scanning-tomographic data acquisition routine of S3DXRD to enable 3D non-destructive characterization of crystalline materials in a home laboratory environment (Zhang & Juul Jensen, 2020 ▸). This method has been proposed and demonstrated at synchrotrons with intense parallel beams in both transmission and 90° reflection geometries (Hofmann *et al.*, 2012 ▸; Hofmann *et al.*, 2013 ▸; Ferreira Sanchez *et al.*, 2014 ▸; Ferreira Sanchez *et al.*, 2015 ▸). This article aims to demonstrate the proposed concept for a laboratory setting, including (i) the principles underlying the method, (ii) its first experimental implementation (encompassing both hardware and data acquisition software), (iii) the development of software for processing the collected data, and (iv) its validation through comparative studies with LabDCT and synchrotron phase contrast tomography (PCT). We demonstrate the feasibility of this approach for high-spatial-resolution characterization. Additionally, we discuss possible future directions for developing the technique into a versatile tool for materials studies, including its potential combination with LabDCT and conventional micro-computed tomography (µCT) to enable multiscale and multimodal analysis.

## 2. Principles of Lab-3DµXRD

**Figure 1:**
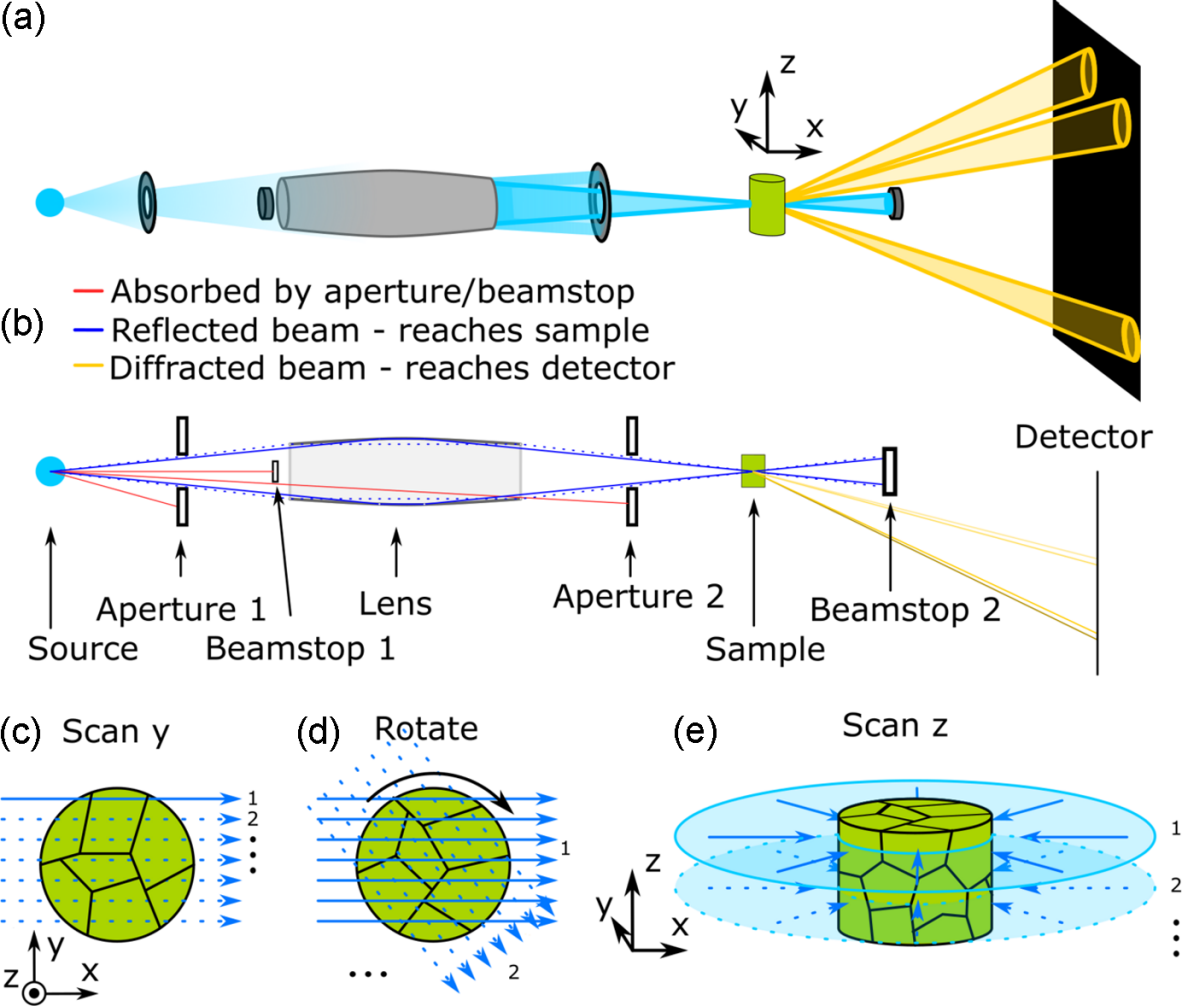
Schematics of (*a*) and (*b*) the Lab-3DµXRD geometry and (*c*)–(*e*) the data acquisition routine. The dashed and solid blue lines in (*b*) represent example incident X-rays with lower and higher energies, respectively. The yellow lines represent diffracted X-rays, with variations in brightness indicating changes in wavelength – brighter lines correspond to shorter wavelengths (*i.e.* higher energies). The angles between the lines, *i.e.* the beam divergence, are significantly exaggerated. Arrows in (*c*) and (*d*) indicate the beam paths into the sample during its translation.

The principles of Lab-3DµXRD are sketched in Fig. 1 ▸. Similarly to LabDCT, a polychromatic X-ray beam is employed to maximize the utilization of the available photons. An X-ray source with a heavy-element target, such as tungsten, is preferred over conventional Cu or Cr sources because the intensity of emitted X-rays increases approximately linearly with the atomic number of the target. In addition, focusing X-ray optics are introduced to further enhance intensity. These optics not only amplify the X-ray flux by concentrating the beam – beneficial for detecting smaller grains –but also limit the field of view illuminated within the sample, which is critical for enabling intragranular characterization. For the characterization of metals such as Al, Ti and steel, it is crucial that the optics can focus high-energy X-rays, ensuring that submillimetre- to millimetre-scale samples can be effectively studied (Seret *et al.*, 2023 ▸).

As with S3DXRD and synchrotron microdiffraction techniques, a detector with a relatively large pixel size is suitable for Lab-3DµXRD. The detector should be positioned in transmission geometry rather than in 90° reflection or 180° back-projection mode. This placement simplifies implementation and maximizes the Lorentz–polarization factor (Warren, 1990 ▸), which is critical for enhancing the intensity of the diffraction signals. Similarly to LabDCT, a beamstop (Beamstop 2 in Fig. 1 ▸) is used to block the transmitted beam, preventing detector saturation and enhancing the detection of the weaker diffraction signals.

With a focused beam, it is straightforward to confine the gauge volume to a narrow channel through the sample. However, achieving full 3D characterization requires recovering depth information, *i.e.* determining the precise location along the gauge channel where diffraction occurs. To address this challenge, we propose a new tomographic data acquisition method to restore depth information and retrieve full 3D crystallographic data, adapting the principles of S3DXRD as originally proposed by Poulsen (2004 ▸):

(1) Laue diffraction images are collected at every step while the sample is scanned along the horizontal direction, *y* in Figs. 1 ▸(*a*) and 1 ▸(*c*), perpendicular to the incoming beam, *x*.

(2) The sample is rotated 360° around the vertical axis (*z*) at equal-angular intervals, and the scan along *y* is repeated after each rotation [see an example in Fig. 1 ▸(*d*)].

(3) By repeating the measurement layer by layer [see Fig. 1 ▸(*e*)], a 3D image of the grain structure can be obtained.

In this way, intragranular information, such as the local crystallographic orientation and strain, in any volume element within the scanned section can be determined by combining the diffraction images collected as the beam passes through this location at different rotation angles (Hayashi *et al.*, 2015 ▸; Henningsson *et al.*, 2020 ▸; Henningsson *et al.*, 2023 ▸).

## 3. Experimental implementation

### 3.1. Material

A beta titanium alloy (Ti-β21S) sample with 300 µm diameter was chosen for this proof-of-concept experiment, as its grain structure was already known from previous high-resolution synchrotron PCT measurements at beamline ID19 of the European Synchrotron Radiation Facility. The microstructure consists of equiaxed grains of the metastable β phase with a body-centered cubic lattice. The sample was annealed at 830°C for 30 min to promote grain growth, yielding an average grain size of 40 µm. A subsequent annealing at 725°C for 15 min formed a thin hexagonal close-packed α phase layer along the grain boundaries. The difference in chemical composition between the two phases provides a detectable electron density contrast, enabling 3D grain shape determination via PCT. The submicrometre-resolution (0.56 µm) 3D grain structure serves as an ideal benchmark for validating Lab-3DµXRD results. More details on the PCT experiment and grain segmentation are provided by McDonald *et al.* (2015 ▸).

### 3.2. Hardware development

To demonstrate the concept, a conventional X-ray tomography instrument (Zeiss Xradia Versa 520), equipped with a flat-panel extension and a LabDCT Pro module, was utilized for the experiment. This system featured a tungsten-target microfocus X-ray source [similar to that used by Lynch *et al.* (2019 ▸)], capable of operating at a maximum energy of 160 keV and a power of 10 W. Pt-coated twin paraboloidal X-ray optics, devised and manufactured by Sigray Inc., were used to focus the X-ray beam. These optics were designed for maximum efficiency at a photon energy of approximately 32 keV. In practice, they are capable of focusing the X-ray beam to a full width at half-maximum of 8.5 µm with a divergence angle of 0.59°. The focal point was located 200 mm downstream from the X-ray source and 30 mm from the exit of the optics (*i.e.* working distance). The resulting focused beam was achromatic, supporting a broad energy range of up to 45 keV. Further details on the characteristics of the optics were given by Seret *et al.* (2023 ▸).

For precise alignment of the optics, a manually adjustable five-axis stage from Thorlabs was employed. To minimize stray radiation, a 400 µm upstream aperture was used, along with an additional 2 mm aperture located downstream of the optics. A Dexela 2315 flat-panel detector, mounted in transmission geometry, was used to record Laue diffraction patterns. The scintillator-based CMOS detector featured a resolution of 1936 × 3064 pixels and a physical pixel size of 75 µm. To block the direct beam and enhance the contrast of the diffracted signals, a 2 mm-thick tungsten beamstop (Beamstop 2 in Fig. 1 ▸) was positioned centrally on the detector.

### 3.3. Data acquisition

For the Lab-3DµXRD experiment, an acceleration voltage of 110 kV and a source power of 10 W were used. These parameters were chosen to optimize the photon flux on the basis of insights from previous studies (Lindkvist *et al.*, 2021 ▸). Following a manual inspection of diffraction images collected at different sample-to-detector distances (*L*_sd_), the flat-panel detector was positioned at *L*_sd_ = 100 mm to ensure that most of the strong diffraction peaks were effectively captured. An exposure time of 105 s was used for each projection, with no detector binning applied.

During the data acquisition, the sample was scanned horizontally in 77 steps with a translation step size of 5 µm at each of the 36 rotation angles, spaced 10° apart. At each rotation angle, a reference image made by averaging five consecutive acquisitions was collected at the end of the translation steps and outside the sample volume to normalize the diffraction images. This helps to compensate for long-term intensity fluctuations and ensures consistent image quality across the dataset. The total scan time for this Lab-3DµXRD experiment was 86 h [accounted for as 105 s × (77 + 5) steps × 36 rotation angles]. Data acquisition was performed using an in-house-developed control framework, designed to orchestrate the imaging system components. For this initial demonstration, only a single slice near the center of the PCT volume was characterized.

To validate the characterized crystallographic orientations, the sample was also scanned using LabDCT with the same detector and mounting setup. The LabDCT measurement was conducted with the same X-ray power and acceleration voltage as Lab-3DµXRD. A total of 181 projections were collected with an exposure time of 60 s during a full 360° rotation, at a source-to-sample distance (*L*_ss_) of 11.5 mm and an *L*_sd_ of 245.8 mm, *i.e.* in a projection geometry (Oddershede *et al.*, 2022 ▸). In addition, a µCT scan was performed to reconstruct the sample shape and facilitate the analysis of the LabDCT data. The total scan time for LabDCT and µCT was about 4 h.

### 3.4. Processing of Lab-3DµXRD data

The process consists of four main steps for each layer: (1) preprocessing raw diffraction images, (2) indexing grain orientations, (3) reconstructing the grain slice morphology and (4) refining the intragranular orientation field.

All four steps were performed in MATLAB with parallelized computing on an HP Zbook Fury 16G10 workstation laptop, with each step detailed in the following subsections and the corresponding processing times summarized in Table S1 of the supporting information. For processing of data from multiple layers, these four steps will be repeated for each layer. However, the results from the first layer can serve as prior knowledge to accelerate the entire process.

#### 3.4.1. Preprocessing for spot detection

**Figure 2:**
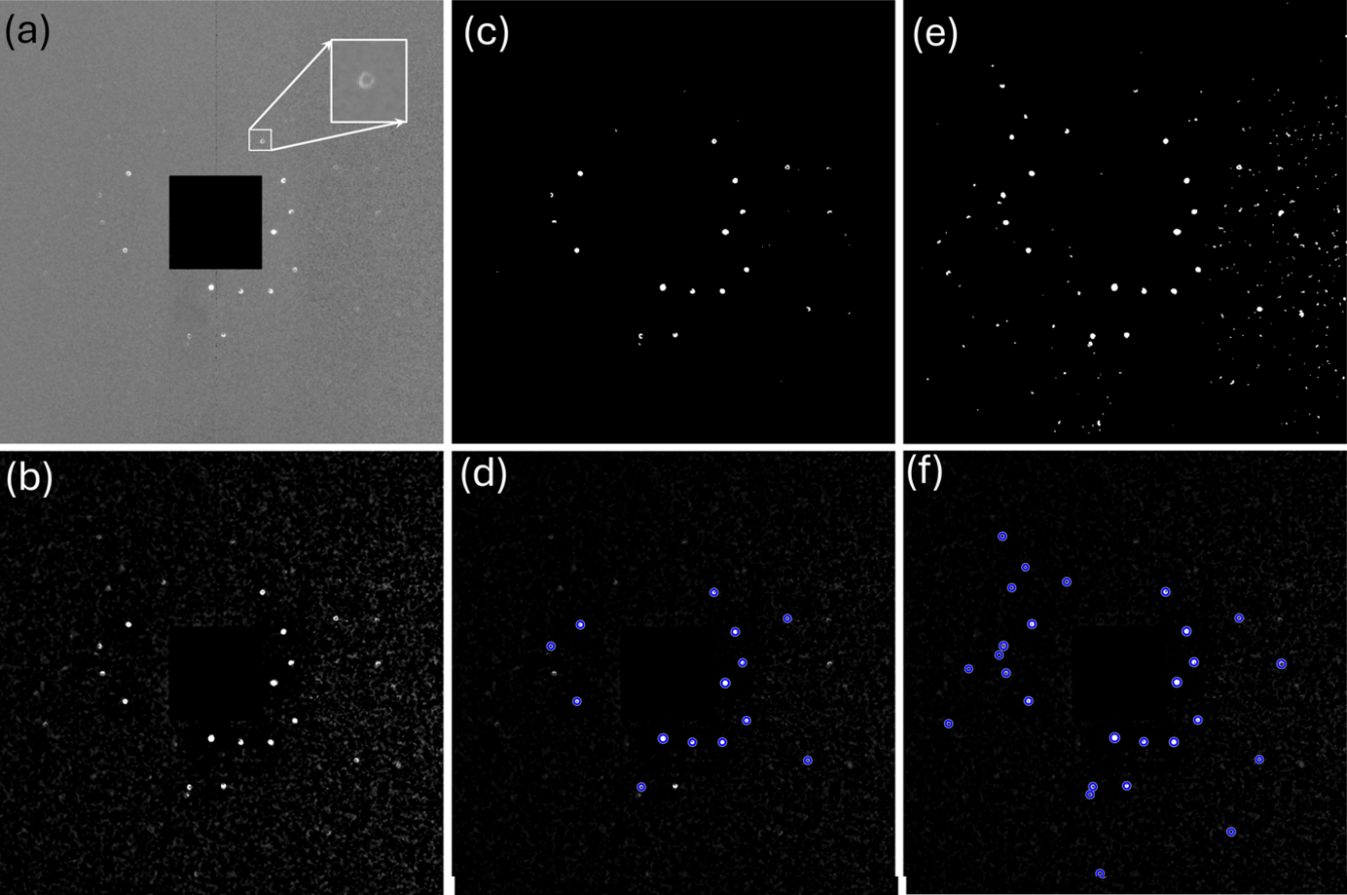
Example of a diffraction image and the processing routine: (*a*) normalized diffraction image; (*b*) after background removal and image processing; (*c*) and (*e*) binarized images of (*b*) using higher and lower threshold values, respectively; (*d*) and (*f*) corresponding detected diffraction spots. Each image represents a physical size of 112.5 × 112.5 mm^2^. The central black region is due to the beamstop (Beamstop 2 in Fig. 1 ▸).

The collected raw diffraction images were processed in groups based on the sample rotation angle. For each group, the raw images were first normalized by dividing by the reference images from the same rotation angle (Lindkvist *et al.*, 2021 ▸). An example of the central region of a normalized diffraction image with many doughnut-shaped diffraction spots is shown in Fig. 2 ▸(*a*). The doughnut shape originates from the incoming beam. The central rays do not interact with the paraboloidal mirrors and therefore are blocked by an internal optics beamstop made of a small tungsten wire (Beamstop 1 in Fig. 1 ▸), preventing them from passing through the optics unfocused (Seret *et al.*, 2023 ▸) [see Fig. 1 ▸(*b*)].

After normalization, intensity fluctuations between images may still be noticeable over time. These variations are caused by X-ray source fluctuations and were corrected using scaling factors determined from the average intensity over a small 100 × 100 pixel area that does not contain visible diffraction spots. A background, determined as the median of the group images collected at each rotation angle, was then subtracted from each intensity-scaled image [see Fig. 2 ▸(*b*)]. To further reduce long-range intensity variations, a Gaussian filter with a sigma of 50 pixels was applied to each of the background-corrected images, and the resulting Gaussian-filtered images were then subtracted from the originals. As a final step in image processing, a non-localized mean filter was applied to suppress any remaining local noise.

Different threshold values were then subsequently applied to segment diffraction spots, with spots smaller than 5 pixels eliminated to minimize noise effects. Binary images segmented using comparatively high (6) and low (2) threshold values are shown in Figs. 2 ▸(*c*) and 2 ▸(*e*), respectively. Though the low threshold yielded a higher number of detected spots, it also resulted in some false detections. As described later, both thresholds were used in the analysis process. Finally, to improve spot center determination, a ring detection algorithm based on the circular Hough transform was employed to identify the doughnut-shaped spots [see Fig. 2 ▸(*d*) and 2 ▸(*f*)].

#### 3.4.2. Orientation indexing

**Figure 3:**
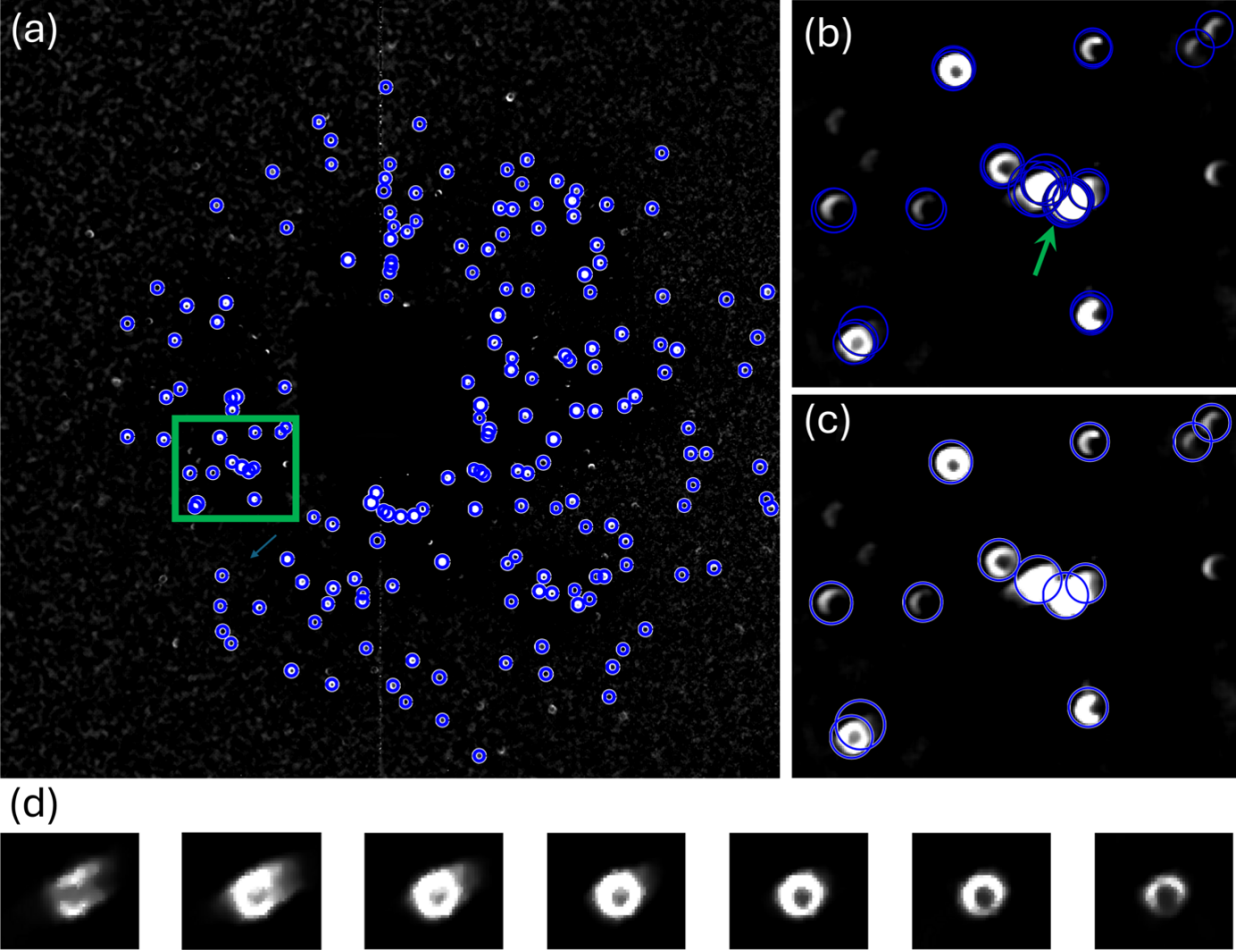
(*a*) Example of a set of diffraction spots, where those appearing at the same location on the detector are merged. (*b*) Magnified view of all detected diffraction spots from images collected at a −180° rotation angle, overlaid onto a sum of all diffraction images; and (*c*) the corresponding merged spot set in the region marked by the green rectangle. (*d*) Diffraction spot, marked by the green arrow in (*b*), appearing at different translation steps.

The centers of the detected rings were used to calculate the diffraction vectors for indexing grain orientations, assuming the origin to be at the center of the scanned volume. To facilitate the indexing, diffraction spots appearing at nearby pixel positions (within a 5 pixel distance in this work) across all individual diffraction images collected during sample translation at the same rotation angle were merged into a single spot. For the present case, this process resulted in 36 sets of diffraction spots corresponding to the 36 rotation steps. An example of the merged diffraction spots for the first rotation angle is shown in Fig. 3 ▸. This merging process significantly reduced the number of diffraction spots for orientation indexing, which was done using the laboratory dictionary- based branch-and-bound algorithm (LabDBB) (Zhang & Lindkvist, 2025 ▸), adapted to the new diffraction geometry used in this work.

The LabDBB algorithm operates by comparing experimental diffraction vectors with theoretical diffraction vectors, calculated from a predefined orientation dictionary. Candidate orientations from the dictionary, known as orientation branches, are determined when a sufficient number of theoretical diffraction vectors from an orientation match experimental vectors within a specified upper-bound deviation angle, defined by the dictionary branch resolution. Once a candidate orientation is identified, it is refined to determine its validity on the basis of additional threshold criteria, such as the number of matched diffraction vectors and their angular deviation. Note that, in experiments using a polychromatic X-ray beam, the exact photon energies corresponding to the diffraction spots are not known. As a result, only normalized diffraction vectors are considered during orientation matching. This approach ensures accurate and efficient determination of grain orientations within the scanned sample layer. Further details about the LabDBB algorithm are given by Seret *et al.* (2022 ▸) and Zhang & Lindkvist (2025 ▸).

To accelerate the indexing process and improve accuracy, a two-step indexing approach was performed. In the first step, diffraction spots detected using the higher threshold were utilized to index the initial grain orientations. This ensured a focus on strong and clearly defined diffraction spots. The indexed orientations were then applied to fit the detector geometry using the method described by Fang *et al.* (2022 ▸), after which they were further refined. In the second step, diffraction spots uniquely assigned to each indexed orientation from the higher threshold set were removed from the lower threshold set. This step effectively filtered the dataset by eliminating already-indexed spots, allowing the algorithm to focus on the additional diffraction spots detected with the lower threshold. The remaining unassigned diffraction spots were then used for further indexing, incorporating weaker diffraction signals into the analysis.

For the present case, a dictionary branch resolution of 2.5° was used, resulting in 39565 orientations generated with *MTEX* (Bachmann *et al.*, 2011 ▸). For each of the two intensity thresholds, a completeness threshold decreasing from 0.7 to 0.4, in steps of 0.1, was applied to limit the number of candidates requiring further time-consuming orientation fitting at each step. The final indexed orientations required a completeness value of 0.3, with their diffraction vectors deviating by no more than 0.25° from the corresponding theoretical predictions. This completeness threshold was chosen by trial and error, as the value below which the false positive rate increased significantly. The completeness value is defined as the ratio of the observed number of spots to the theoretical number of spots, calculated using the first three low-index {*hkl*} families of a body-centered cubic β-Ti crystal structure, namely {110}, {200} and {112}, and the X-ray energy range 15–45 keV.

#### 3.4.3. Reconstruction of grains and post-processing of assembled grain structure

**Figure 4:**
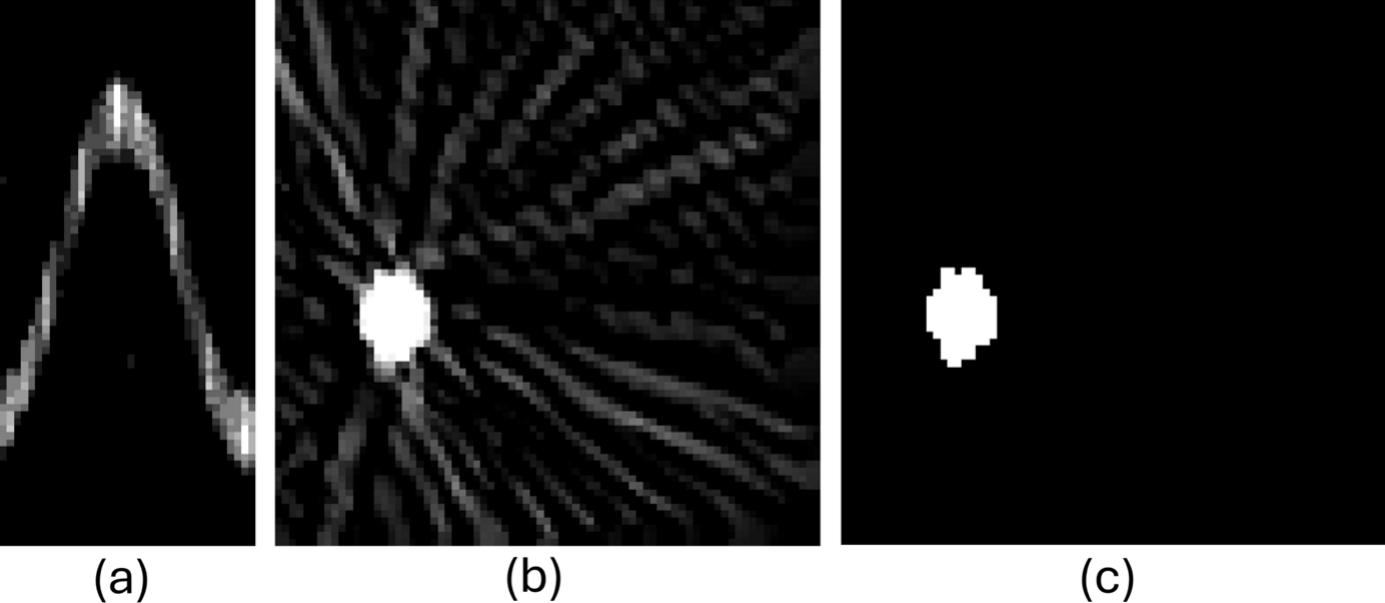
Example illustrating the grain reconstruction process. (*a*) Sinogram showing the number of matched diffraction spots for this grain orientation at each translation step (vertical axis) and each rotation angle (horizontal axis). (*b*) Reconstructed grain using the sinogram in (*a*). (*c*) Segmented grain.

The number of diffraction spots matching each indexed orientation was determined using all individual, unmerged diffraction vectors obtained from each diffraction image at every scan position. This matching process involved comparing diffraction vectors, determined using the first ten {*hkl*} families, within a larger deviation angle of 0.5° and a maximum distance of 10 pixels for the associated spots. A large number of {*hkl*} families were used to ensure the detection of the grains at more rotation angles, while the larger deviation angle was used to account for local variations in spot positions, which can arise from noise and orientation differences across translation steps. The number of matched diffraction spots was then used to create a sinogram for reconstructing the grain shape. An example of such a sinogram is shown in Fig. 4 ▸(*a*). The sinograms were subsequently used to reconstruct grains using a filtered back-projection method. The reconstructed grain using the sinogram in Fig. 4 ▸(*a*) is shown in Fig. 4 ▸(*b*), from which the grain shape was determined using a single-threshold segmentation approach [see Fig. 4 ▸(*c*)].

The grain map within the scanned layer was assembled by merging all the reconstructed grains. Overlapping pixels between grains were resolved by evaluating the pixel completeness values for all candidate grains at these pixels. The pixel completeness value was defined as the ratio of the observed number of spots from all images collected while the beam passed through the pixel and the theoretical number of spots expected for the candidate grain. Ultimately, each pixel was assigned to the grain with the highest completeness value. Additionally, unindexed pixels were assigned an orientation corresponding to the neighboring grain with the highest completeness value for the given pixels.

#### 3.4.4. Refining the intragranular orientation field

When the grain structure has been reconstructed, the orientation of each grain can be further refined to reveal intragranular orientation variations by analyzing diffraction images collected as the beam passes through a given pixel, *i.e.* similar to the point-wise fitting in S3DXRD (Hayashi *et al.*, 2015 ▸; Hayashi & Kimura, 2023 ▸). To enhance orientation accuracy, more spots from up to ten {*hkl*} families were used, and a stricter angular deviation of 0.25° was applied for filtering diffraction spots. For this analysis, the actual pixel position was included in the refinement.

### 3.5. Validation

To validate the orientations determined by Lab-3DµXRD, the LabDCT data were processed using *GrainMapper3D*, developed by Xnovo Technology ApS. A standard LabDCT spot segmentation routine was applied, incorporating rolling median background noise correction followed by a Laplacian of Gaussian based filtering method to generate binary images for grain reconstruction. A fast geometric indexing algorithm (Bachmann *et al.*, 2019 ▸) was used to reconstruct the 3D volume of a total height of 400 µm. Reconstruction parameters were set to standard values, consistent with those used by Oddershede *et al.* (2022 ▸). Grains were identified from neighboring regions with a maximum misorientation of 0.2°.

The reconstructed 3D grains from synchrotron PCT reconstruction were downsampled by a factor of 3 to give the same effective voxel size as the absorption mask reconstruction of the LabDCT scan (*i.e.* 1.7 µm). The reconstructed PCT volume was then segmented into individual grains, providing shape, size and location information but not their orientations. Hence, the PCT grains were registered to the Lab-3DµXRD results by minimizing the pairwise distances between the center-of-mass positions of the two maps.

## 4. Results

### 4.1. Lab-3DµXRD results

**Figure 5:**
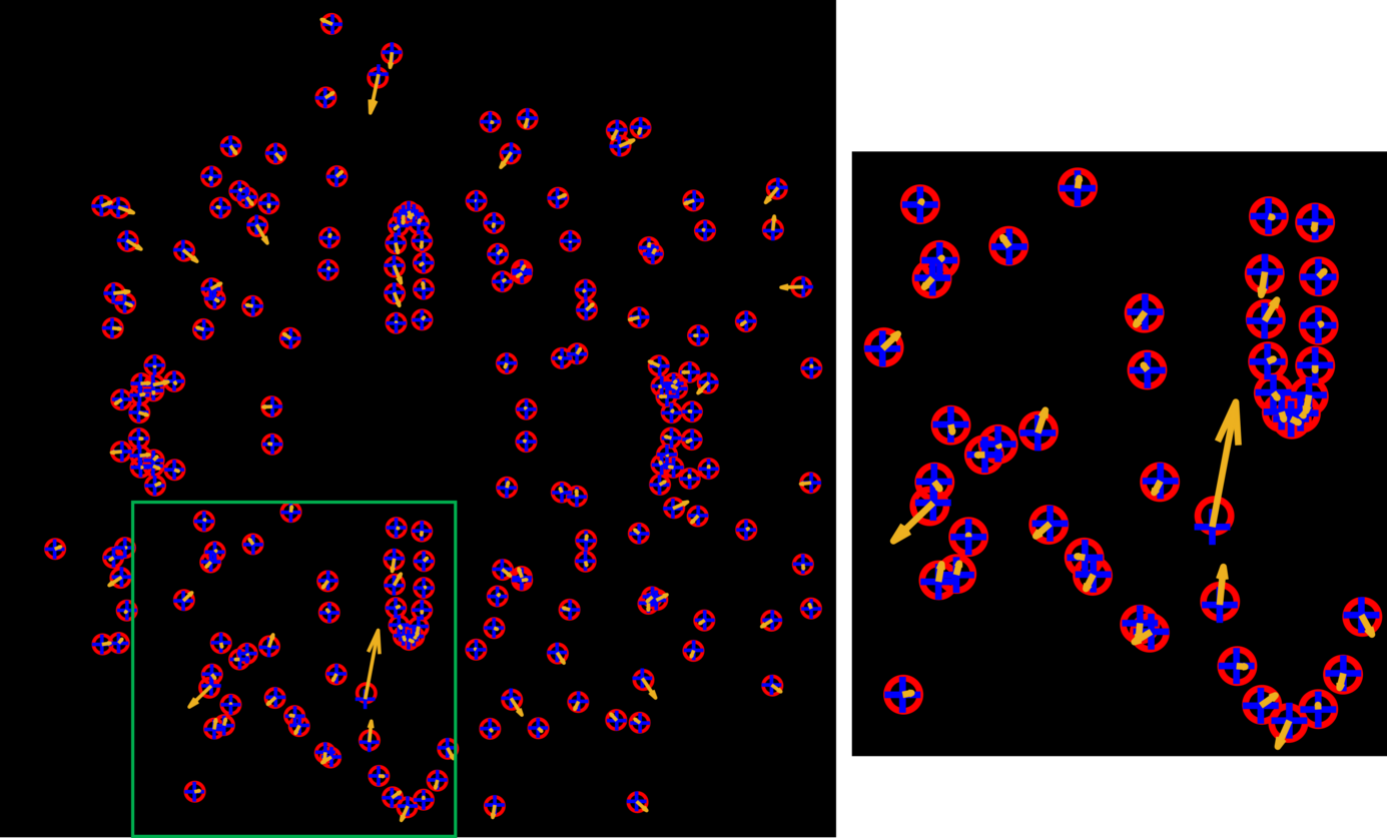
Comparison between experimentally observed, merged diffraction spots (shown as red circles) and their theoretical positions (marked by blue crosses) for a relatively large grain. This image consolidates all the diffraction spots from the first ten {*hkl*} families observed in the 36 integrated diffraction images. The orange arrows indicate the direction and relative magnitude of the displacement between each pair of spots. The image on the right shows a magnified view of the region marked by the green rectangle.

In total, 73 grain orientations were determined from the LabDBB analysis of the Lab-3DµXRD data, among which one orientation did not result in satisfactory grain-shape reconstructions. The completeness value for this orientation was 0.30, and it was therefore considered a false positive and excluded from further analysis. For the largest grain observed within the scanned layer [with an equivalent circular diameter (ECD) of 80 µm], approximately 100 and 250 diffraction spots were observed for the first three and ten {*hkl*} families, respectively (see Fig. 5 ▸). In contrast, only 34 and 50 spots, respectively, are observed for the smallest grain (ECD ≃ 10 µm). This reduction occurs primarily because smaller grains produce weaker diffraction signals that often fall below the background noise threshold. Nevertheless, some falsely detected spots from noise may occasionally be included in the indexing process: see, for example, those marked by the large orange arrows in Fig. 5 ▸.

**Figure 6:**
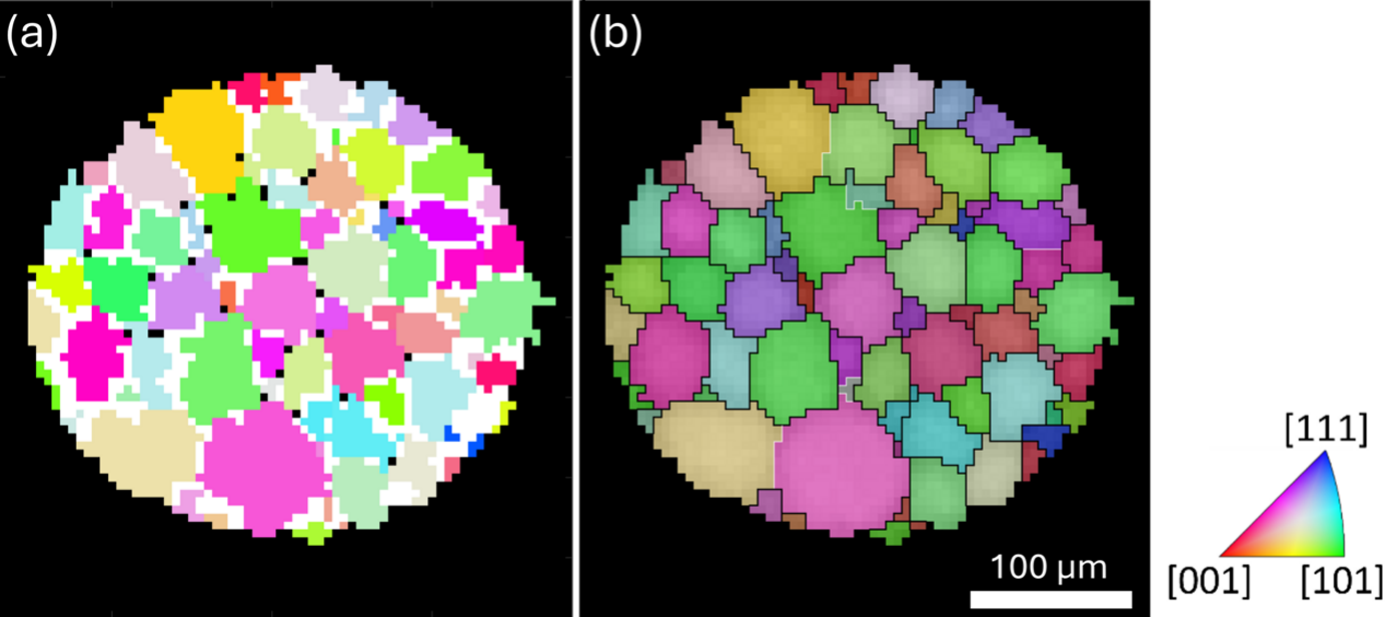
Reconstructed grain map. (*a*) Segmented grains assembled directly. The white and black pixels inside the sample volume represent overlapped and unindexed pixels. (*b*) Grain map after processing. White and black lines indicate boundaries with misorientation angles larger than 2° and 15°, respectively. The brightness of each pixel represents its completeness value.

The raw reconstructed grain map is shown in Fig. 6 ▸(*a*). A pixel size of 5 µm was used for the reconstruction, matching the scanning step size. Most of the overlapping and unindexed pixels are located at the grain boundaries, suggesting a representative reconstruction of the grains. However, two grains completely overlapped with other grains and were eliminated during data cleaning. These two grains are likely to be false positives, resulting from incorrectly detected diffraction spots caused by detector noise. A cleaned map after resolving overlapped and unindexed pixels is shown in Fig. 6 ▸(*b*), where the completeness value for each pixel is visualized by the brightness of the pixel. A total of 70 grains are present in the scanned layer, with most of the grain boundaries being high-angle boundaries. The limited number of false positives (3 out of 73 indexed grains) confirms the appropriateness of the selected indexing completeness threshold.

### 4.2. Comparison with LabDCT and PCT

**Figure 7:**
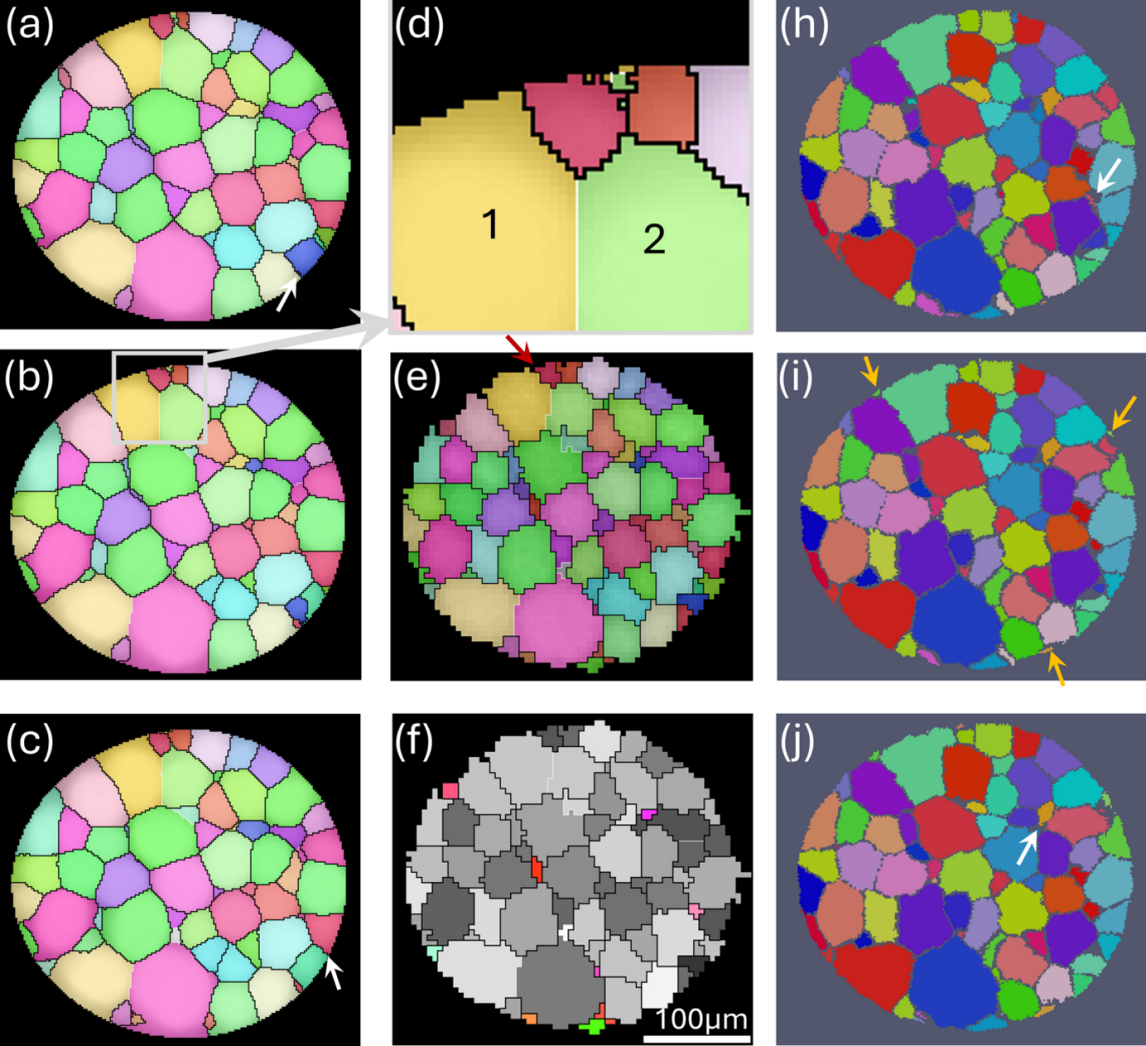
Comparison of LabDCT, Lab-3DµXRD and PCT. (*a*)–(*c*) Three sequential slices reconstructed using LabDCT with a voxel size of 2 µm. (*d*) Magnified view of the region marked by the gray rectangle in (*b*). (*e*) Lab-3DµXRD result reconstructed with a voxel size of 5 µm. (*f*) Grains indexed by Lab-3DµXRD but not LabDCT, highlighted in colors. (*h*)–(*j*) Three sequential slices showing the grain structure segmented on the basis of PCT results with a voxel size of 1.7 µm. The grains in the PCT data are randomly colored.

The Lab-3DµXRD results are compared with the LabDCT and PCT results in Fig. 7 ▸. Three sequential slices from the scanned volume in both LabDCT and PCT were selected for a comprehensive comparison. A visual comparison of the three slices in the PCT data with the Lab-3DµXRD slice reveals that the middle slice [Fig. 7 ▸(*i*)] provides the best match, as some grains found in the Lab-3DµXRD slice are missing in the other two PCT slices; two examples are marked by the white arrows. A similar conclusion can be drawn for the LabDCT data, where the middle slice, Fig. 7 ▸(*b*), provides the best match. Note that since the missing grains in the two neighboring slices (at opposite sides) are close in (*x*, *y*) coordinates, the mismatch between these slices and the Lab-3DµXRD slice cannot be attributed to sample volume tilt between different measurements. This is particularly true for the LabDCT data, as they were collected using the same sample mounting as Lab-3DµXRD.

Since the synchrotron PCT data were acquired with a finer resolution and absorption contrast is sensitive to the density difference of the alpha phase precipitated at the grain boundaries, the PCT-segmented grain structure is considered the ‘ground truth’ for the 3D grain shape in this study. A close comparison of individual grains suggests that Lab-3DµXRD was able to detect nearly all the grains present in the best-matched PCT slice, except for the three small grains marked by the orange arrows in Fig. 7 ▸(*i*). These grains are located at the surface of the sample and are less than 7 µm in size on this slice. Compared with LabDCT, Lab-3DµXRD detects nine additional grains with sizes around 10–20 µm, as shown in Fig. 7 ▸(*f*). For the remaining matched grains, the misorientation between the Lab-3DµXRD and LabDCT data is below 0.07° for all but one grain, which showed a misorientation of 0.12°, documenting the high precision in Lab-3DµXRD orientation determination.

In addition, several small grains or voxels indexed by LabDCT, such as those in the region marked by the gray rectangle in Fig. 7 ▸(*b*) and magnified in Fig. 7 ▸(*d*), are not indexed by Lab-3DµXRD. The orientations of these voxels match those of the two next-neighboring large grains (the yellow grain 1 and green 2). Since these LabDCT grains are also absent in the corresponding PCT slice, they are considered false positives, likely resulting from the space-filling algorithm and a small voxel size used during indexing.

Finally, Lab-3DµXRD detects one small grain, marked by the red arrow in Fig. 7 ▸(*e*), which is not present in the PCT data. Since this grain is also detected by LabDCT and the overall shape of this grain, together with the neighboring yellow grain 1, matches the corresponding large turquoise grain in the PCT data, it is considered correctly identified. The misorientation of the boundary between these two grains is 45.8°〈0.19, −0.08, 0.98〉, which does not belong to any low-index coincident site lattice boundaries. It is unknown why it was not properly detected by PCT.

### 4.3. Intragranular orientations

**Figure 8:**
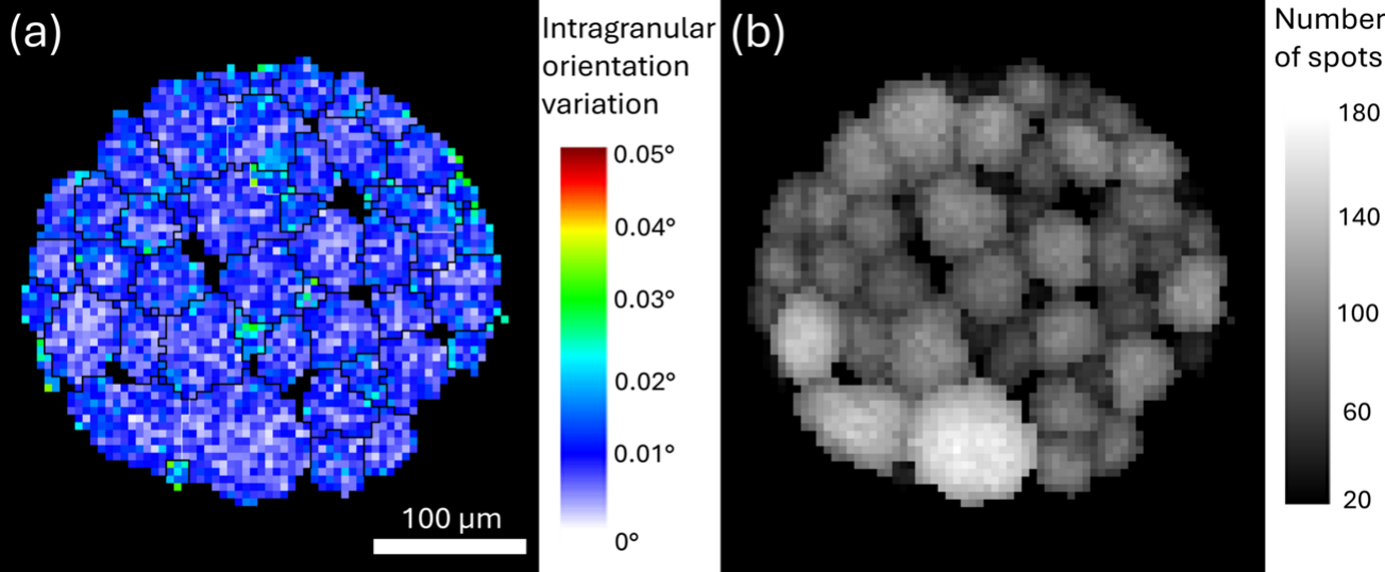
(*a*) Intragranular orientation variation, calculated as the deviation from the average orientation of the grain, for the 50 largest grains. (*b*) Number of detected spots for each pixel.

The intragranular orientation quantified as the deviation angle of each pixel to the grain average orientation is shown in Fig. 8 ▸. Since the accuracy is directly related to the number of detected spots, only the 50 largest grains are shown here. A maximum deviation of ∼0.03° from the average orientation was detected in some grains, particularly among a few surface grains. Such small orientation changes may arise from surface damage on the sample [see, for example, the elongated spot in Fig. 2 ▸(*d*)] but could also result from noise, as they are most likely to be associated with a reduced number of detected spots close to grain boundaries (and in small interior grains). The average misorientation between neighboring pixels within individual grains is 0.006–0.008°. Considering the thermomechanical processing history of the present sample with a very well annealed grain structure, this level of orientation variation (∼0.01°) is likely to represent the orientation uncertainty of the present dataset. This low uncertainty is attributed to the small ratio of detector pixel size to detector distance, as well as the large number of diffraction spots involved in the setup.

## 5. Discussion

### 5.1. Feasibility of Lab-3DµXRD

The above analysis and comparison clearly demonstrate the feasibility of Lab-3DµXRD for 3D grain mapping. Specifically, the proposed rotation and scanning strategies can provide depth-resolved grain orientation information. With the white beam employed in Lab-3DµXRD, the number of rotation angles can be significantly reduced — by a factor of ∼10 compared with synchrotron S3DXRD (Hayashi *et al.*, 2015 ▸), which uses a focused monochromatic beam. This is because a single Laue diffraction image can contain more than ten diffraction spots from the same grain, whereas in S3DXRD, there is rarely more than one spot per rotation step per grain. Additionally, in S3DXRD, a unique (*hkl*) plane can produce only two spots during a full 360° rotation, whereas in Lab-3DµXRD, a unique (*hkl*) plane can produce many more spots from different X-ray energies, depending on the number of rotation steps and the direction of the corresponding diffraction vector (see Fig. 5 ▸). Furthermore, the continuous X-ray spectrum eliminates the need for sample rocking during data acquisition, simplifying the overall process.

Besides the Pt-coated twin paraboloidal capillary optics used in the present setup, other types of focusing optics, such as polycapillary optics (Lynch *et al.*, 2019 ▸) and multilayered supermirrors (Joensen *et al.*, 1994 ▸), may also be used. In that case, the front optics beamstop (Beamstop 1 in Fig. 1 ▸), which blocks the central part of the beam, can be omitted, resulting in diffraction spots with a full-circle shape. However, to the best of the authors’ knowledge, the present Pt-coated capillary optics outperform polycapillary optics in terms of intensity gain, focal spot size, beam achromatism and the X-ray energy range of the focused beam (Seret *et al.*, 2023 ▸). The practical implementation of multilayered supermirrors in a laboratory setting has yet to be demonstrated.

In this first implementation, a sample rotation axis perpendicular to the incoming beam is chosen. While this is the most common setup for tomographic data acquisition, a rotation axis inclined at a non-perpendicular angle can also be used for implementing Lab-3DµXRD, as recently demonstrated by Kim *et al.* (2023*b* ▸). This inclined rotation axis setup may be beneficial for improving the spatial resolution along the vertical direction.

### 5.2. Advantages of Lab-3DµXRD

Compared with existing laboratory-based techniques, Lab-3DµXRD offers several distinct advantages. Lab-3DµXRD demonstrates a capability in detecting small grains (<20 µm), which is beneficial for gathering grain neighborhood and topology information, and thus for studying grain growth. This enhanced detection capability arises from the novel use of paraboloidal optics, which provide two key advantages: (1) significantly increased X-ray intensity (Seret *et al.*, 2023 ▸) and (2) a narrow ‘1D’ gauge volume/channel that precisely interrogates the microstructure. The enhanced X-ray intensity enables stronger diffraction signals for spot detection, while the focused 1D beam ensures that the diffraction signal is linearly proportional to the grain size (*R*). In contrast, the LabDCT signal is proportional to the entire 3D volume (*R*^3^). The more balanced spot intensities from grains of different sizes allow Lab-3DµXRD to operate with longer exposure times to resolve small grains without detector saturation from the large grains. Furthermore, since diffraction spots always originate from a local volume at the focal point, the total number of spots per image is typically low (around 20–30 in the present case). This minimizes spot overlap. Consequently, grain indexing and reconstruction can be relatively simple.

More importantly, Lab-3DµXRD can provide intragranular orientation information. Compared with the Laue focusing effect, micro-beam Laue diffraction is less sensitive to lattice defects, implying that Lab-3DµXRD is capable of studying plastic deformation. For example, previous results show that Lab-µXRD, equipped with a photon-counting detector (see more details below), can detect diffraction signals from additively manufactured AlSi10Mg alloys (Zhang *et al.*, 2024 ▸), although the crystallographic orientation could not be determined from a single diffraction image. With Lab-3DµXRD, more diffraction spots would be captured during sample rotation, enabling the determination of the orientation field within grains. Such a study is planned for the near future.

### 5.3. Future Lab-3DµXRD method development directions

In principle, improved grain detection can enhance the accuracy of grain boundary positioning. A practical approach to achieving this is to develop a forward-simulation-based reconstruction method, similar to LabDCT (Bachmann *et al.*, 2019 ▸), capable of resolving the grain structure at a voxel size smaller than the selected translation step size (5 µm in this work). A recently demonstrated super-resolution strategy based on the superposition of X-ray beam trajectories (Kim *et al.*, 2023*a* ▸) could also be explored as a potential enhancement to spatial resolution.

A significant issue of the present Lab-3DµXRD is the long data acquisition time needed for mapping a representative 3D volume. Although the current experimental setup, which utilizes a conventional flat-panel detector, has extended the detection limit to 10 µm, further improvement remains constrained by the detector’s sensitivity and dynamic range. Consequently, detecting even smaller grains (<10 µm) remains challenging with the present setup.

**Figure 9:**
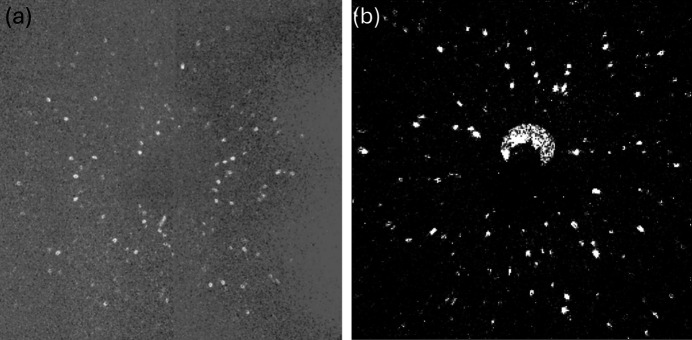
Diffraction images collected from an iron powder sample (but not from the same location) using (*a*) the same flat-panel detector with an exposure time of 170 s and (*b*) a photon-counting detector (Advacam ADVAPIX TPX3, 256 × 256 pixels and 55 µm pixel size, placed at an *L*_sd_ of 14 mm) with an exposure time of 1 s. The intensities are after normalization using a reference image collected under the same conditions but without the sample.

Further improvement of the efficiency of the optics, as suggested by Seret *et al.* (2023 ▸), to eliminate both long-range and short-range defects of the mirror surface could reduce the focal spot size and increase the X-ray intensity. More importantly, the adoption of advanced photon-counting detectors (Zhang *et al.*, 2024 ▸), which offer higher sensitivity, an improved dynamic range and reduced noise, presents a viable solution to further decrease acquisition time and enhance the detection of smaller grains. A preliminary test on a pure iron sample has demonstrated that a realistic gain factor of ∼200 in exposure time is achievable with an Advacam ADVAPIX TPX3 photon-counting detector, as illustrated in Fig. 9 ▸. The sample was prepared from a powder with a nominal particle size of 5–8 µm and sintered at 850°C for 0.5 h in a borosilicate glass tube with inner and outer diameters of 80 and 100 µm, respectively, resulting in a relative density of about 75%. The exact grain size after sintering is unknown. However, on the basis of the high number of detected diffraction spots (about 80), it is likely that more than 10 grains were present within the illuminated volume channel. With increased exposure time, it is expected that the high photon sensitivity of the detector will enable the detection of grains as small as 5 µm. By combining this enhanced detector performance with a 10–100× gain in X-ray flux – achievable using, for example, an intense X-ray source from a liquid metal jet anode (David *et al.*, 2004 ▸), linear accumulation X-ray sources (Yun *et al.*, 2017 ▸) or a tabletop synchrotron light source (Yamada *et al.*, 2014 ▸) – the exposure time could be reduced by an order of magnitude or more. Together, this advancement could enable Lab-3DµXRD to achieve 3D mapping of grains as small as 2–5 µm and of samples with a high defect content, such as additively manufactured materials (Zhang *et al.*, 2024 ▸) in laboratory settings.

Besides the hardware and software improvements, further optimization of experimental parameters, such as the maximum X-ray energy and power, scanning step size, number of rotation angles and detector distance, is necessary. Typically, increasing the input power can enlarge the X-ray source size, which in turn leads to an increase in focal spot size. However, this enlargement may not significantly compromise the ring shape or symmetry of the focused beam with the present focusing optics. As a result, its impact on the diffraction spot shape could remain minimal. To quantify this effect, a ray-tracing simulation, such as a Monte Carlo method (Knudsen *et al.*, 2013 ▸), could be conducted. Moreover, the feasibility of the technique for other material systems, such as Al and steel, with varying sample sizes, numbers of grains, absorption rates and defect contents, should be explored.

Furthermore, Lab-3DµXRD could be combined with existing techniques such as LabDCT and µCT, enabling multiscale and multimodal characterization of materials. For example, Lab-3DµXRD could be combined with LabDCT to study recrystallization, where Lab-3DµXRD is used to characterize nuclei and the deformed microstructure, while LabDCT tracks the subsequent grain growth (Zhang & Ludwig, 2024 ▸). For alloys containing second-phase particles, it could then be combined with µCT to study particle-stimulated nucleation (Knipschildt-Okkels *et al.*, 2025 ▸), where µCT provides information on particle distribution and type. This approach could also be extended to studies of plastic deformation, where LabDCT and µCT capture the overall grain structure and phase distribution of the sample, while Lab-3DµXRD tracks the local microstructural evolution during deformation (Yu *et al.*, 2021 ▸). For samples containing second-phase particles, local plastic strain could further be quantified through particle displacement analysis using *in situ* µCT data (Kobayashi *et al.*, 2022 ▸). In addition, multimodal experimental datasets could be compared or coupled with 4D multimodal digital twin datasets to advance modeling tools and provide unprecedented insight into the evolution of material behavior across time and length scales (Marano *et al.*, 2024 ▸; Li *et al.*, 2024 ▸). Altogether, these advancements would significantly expand the scope of materials research accessible at laboratory-scale facilities, bridge the gap between laboratory and synchrotron capabilities, and open a whole new arena for in-house 3D characterization.

## 6. Conclusions

Lab-3DµXRD has been successfully implemented in a con­ventional X-ray CT system using Pt-coated twin paraboloidal capillary X-ray focusing optics and in-house-developed data acquisition and processing software. By comparing with LabDCT and synchrotron PCT, it is documented that grains in the size range 10–20 µm can be well reconstructed by Lab-3DµXRD with an orientation uncertainty as low as ∼0.01°. This capability provides excellent grain neighborhood information, albeit with poor temporal resolution. With commercially available advanced photon-counting detectors and more intense X-ray sources, Lab-3DµXRD is expected to become capable of detecting grains as small as 2–5 µm and of characterizing deformed materials. Since Lab-3DµXRD shares most of its hardware components with LabDCT, integrating both techniques within a single X-ray CT system is feasible, enabling comprehensive multiscale and multimodal characterization for gaining comprehensive insights into material behavior and properties.

## Supplementary Material

Supplementary table. DOI: 10.1107/S1600576725007587/xx5077sup1.pdf

## Data Availability

Research data and code are available upon reasonable request.
