# Supplementary material for: Laboratory three-dimensional X-ray micro-beam Laue diffraction
Source: J Appl Crystallogr. 2025 Sep 24;58(Pt 5):1742–52. doi: 10.1107/S1600576725007587 (PMC12502863; doi:10.1107/S1600576725007587)
Supplement: Supplementary file 1 [file j-58-01742-sup1.pdf]

JOURNAL OF APPLIED CRYSTALLOGRAPHY

Volume 58 (2025)

Supporting information for article:

Laboratory three-dimensional X-ray micro-beam Laue diffraction

Yubin Zhang, Anthony Seret, Jette Oddershede, Azat Slyamov, Jan Kehres, Florian Bachmann, Carsten Gundlach, Ulrik Lund Olsen, Jacob Bowen, Henning Friis Poulsen, Erik Lauridsen and Dorte Juul Jensen

Laboratory Three-dimensional X-ray Micro-beam Laue Diffraction

Yubin Zhang[1], Anthony Seret[1,2], Jette Oddershede[3], Azat Slyamov[3], Jan Kehres[4], Florian Bachmann[3], Carsten Gundlach[4], Ulrik Lund Olsen[4], Jacob Bowen[3], Henning Friis Poulsen[4], Erik Lauridsen[3], and Dorte Juul Jensen[1]

[1]Technical University of Denmark, Department of Civil and Mechanical Engineering, 2800 Kgs. Lyngby, Denmark
[2]Observatoire de la Côte d'Azur, CNRS, Laboratoire Lagrange, Université Côte d'Azur, CS 34229 - F 06304 NICE Cedex 4, France
[3]Xnovo Technology ApS, 4600 Køge, Denmark
[4]Technical University of Denmark, Department of Physics, 2800 Kgs. Lyngby, Denmark

Table 1: Processing times for each step of the analysis using an HP Zbook Fury 16G10 workstation laptop (Intel(R) Core(TM) i9-13950HX CPU (24 cores), 32 GB RAM) with parallelized computing.

**Processing Step**	**Time**
Preprocessing	2.2 h
Indexing	17 min
Grain reconstruction	27 s
Orientation refinement	39 min
